# Optic Nerve Ultrasound Evaluation in Idiopathic Intracranial Hypertension

**DOI:** 10.3389/fmed.2022.845554

**Published:** 2022-03-01

**Authors:** Maddalena De Bernardo, Livio Vitiello, Ilaria De Pascale, Luigi Capasso, Palmiro Cornetta, Nicola Rosa

**Affiliations:** ^1^Eye Unit, Department of Medicine, Surgery and Dentistry, “Scuola Medica Salernitana, ” University of Salerno, Salerno, Italy; ^2^Corneal Transplant Unit, Azienda Sanitaria Locale Napoli 1, Naples, Italy; ^3^Eye Unit, “Maria SS Addolorata” Hospital, Azienda Sanitaria Locale Salerno, Salerno, Italy

**Keywords:** idiopathic intracranial hypertension, optic nerve, optic nerve sheath diameter, ONSD, ultrasonography

## Abstract

Idiopathic intracranial hypertension (IIH) is a disease with a heterogeneity of possible causes, which needs to be quickly diagnosed. Ocular ultrasonography could be considered a useful tool to diagnose this condition in a fast and non-invasive way. In fact, Karl Ossoinig had already proposed this diagnostic tool in the 1970s for the evaluation of intracranial pressure changes under several pathological conditions, including idiopathic intracranial hypertension. The aim of this review is to analyze scientific articles published in the last 30 years concerning the use of ocular ultrasonography to assess optic nerve indices in patients with idiopathic intracranial hypertension. Specifically, 15 published articles found in PubMed database were included and analyzed in the present review. Our conclusion suggests that ocular ultrasonography is a reliable diagnostic technique to be utilized in all the cases of suspected raised intracranial pressure. To obtain the best possible accuracy and precision in the least invasive way, standardized A-scan seems to be the best choice.

## Introduction

An increase in pressure within the skull is known as intracranial hypertension. Posttraumatic damage, brain tumors, and other space-occupying lesions, such as subdural or epidural hematomas, brain abscesses, and cerebral hemorrhage can produce increased intracranial pressure (ICP) (1). In addition, intracranial hypertension can also be caused by venous blood flow obstructions, such as venous sinus thrombosis and hypoxia-ischemia, increased cerebral volume, inflammatory processes and infections, and hepatic or hypertensive encephalopathy (1).

Sometimes, the reasons for the increase in ICP are unknown (1). In such cases, the diagnosis of idiopathic intracranial hypertension (IIH) or pseudotumor cerebri (PTC) is made (2). It typically affects young obese women of child-bearing age (3), while there is no sex predilection in prepubertal age. Increased cerebrospinal fluid (CSF) production and/or reduced CSF absorption are among the etiologic hypotheses for such a disease. However, for both mechanisms, no proven causes have been found (hence the term *idiopathic* intracranial hypertension) (3). IIH symptoms and signs are progressive daily-occurring non-pulsating headache associated or not with nausea, papilledema, enlarged blind spot, visual field defect or complete visual loss, increased CSF pressure (>200 mmH_2_O in the non-obese, >250 mmH_2_O in the obese) (2). In addition to the symptoms mentioned ahead and the absence of typical lesions that can cause an ICP increase, diagnostic criteria also include bilateral papilledema and normal or slit ventricles on CT scan of the head.

Intraparenchymal probes and intraventricular catheterization, together with lumbar puncture (LP), are considered to be the gold standard approach for measuring ICP (4). In particular, LP is considered to be crucial not only for the diagnosis but also for the treatment of this condition (2). However, in the management of intracranial hypertension, osmotic therapy and, in selected cases, neurosurgery are the main therapeutic approaches (5).

Even though the increase of the optic nerve sheath diameter (ONSD), measured with standardized echography, has been proven to diagnose the presence of increased ICP since the last century (6, 7), its use has been spread only in the last years. Safety, non-invasiveness, and the possibility to examine the patients at bedside are the main advantages of this diagnostic tool, while the major disadvantage is the necessity of well-trained operators (8–11).

Standardized A-scan was employed to measure ONSD for the first time in 1970s by Karl Ossoinig (6, 7), This technique utilizes an 8 MHz non-focused probe, with a special S shaped amplification, which easily showed discerned high-reflective spikes at the interface between the arachnoid and the subarachnoid fluid, providing more accurate and precise ONSD measurements compared to B-scan (12–14).

Considering the growing interest in this diagnostic method and its countless medical applications, the aim of this review to determine the usefulness of ocular ultrasonography in both diagnosing and following up patients with IIH, through the analysis of the published literature on this challenging topic in the last 30 years.

## Materials and Methods

PubMed medical database and Scopus and Web of Science were used to perform our search utilizing words like “ocular ultrasonography” and “idiopathic intracranial hypertension” in our search strings.

January 1990 was chosen as the earliest date of publication and August 2021 was chosen as the latest date of publication.

This review included only full articles, case reports, and case series related to optic nerve ultrasonography assessment for identifying IIH. Additional inclusions were found by manually searching references from the original articles.

## Results

Our first search found 68 articles, 53 of which were excluded after further analysis because they were not directly connected to the issue under discussion. Finally, 15 were included in the present review.

Several studies demonstrated the efficacy of ONSD ultrasound evaluation in detecting raised ICP and identifying patients with suspect IIH. In particular, 5 articles evaluated transorbital B-mode sonography measurements in association to LP, 2 articles talked about ONSD measurements in the Emergency Department, 2 articles evaluated ocular B-mode ultrasound in association with measurement of color-doppler indices of ophthalmic vessels, 4 articles evaluated ONSD measurements as a follow-up of different therapeutic strategies, 1 article was about the diagnosis of IIH combining ONSD measurements and MRI pit/sella ratio, while in only 1 article, authors performed A-scan ultrasonography to appraise patients with IIH.

### ONSD in IIH Patients Before and After Lumbar Puncture

Transorbital B-mode sonography to assess ONSD in patients with IIH that required LP was performed in several articles, such asin a case report by Singleton et al. (15) 30 min before and 30 min after a therapeutic LP, in another case report by Hassen et al. (16) during LP, in a case-control study by Bäuerle et al. (17) before and 1 day after therapeutic LP, in a case-control study by Del Saz-Saucedo et al. (18) before LP and 30 min after it, in a case-control study published by Kishk et al. (19) before and following LP.

In all these articles, patients with IIH had a significantly enlarged ONSD before LP, with a bilateral decrease of ONSD and an improvement of symptoms after LP. Therefore, according to these findings, ultrasound ONSD measurement could be considered a good surrogate marker for changes in the ICP, as it was able to reproduce in real-time intracranial pressure changes.

### ONSD in Patients With IIH in the Emergency Department

Optic nerve sheath diameter ultrasound assessment could be useful also in the ED, considering that emergency physicians are frequently required to identify and triage patients with increased ICP, and IIH is a possible cause that should be considered.

Huo et al. (20) emphasized the utility of a point-of-care ultrasound in the ED to diagnose IIH rapidly and less invasively. Equally, Hassen et al. (21) suggested the practice of bedside ONSD ultrasound assessment in ED not just to quickly diagnose and identify patients with IIH but also to differentiate headache associated with IIH exacerbation from post-lumbar puncture headache (PLPH), a complication due to a fast decrease of ICP (if the headache is due to disease progression, ONSD would be enlarged, whereas PLPH would not).

### ONSD and Color-Doppler Indices of Ophthalmic Vessels in Patients With IIH

Color doppler ultrasonography has been used to evaluate the ophthalmic veins indices with conflicting results (22, 23).

Ebraheim et al. (22) in their study stated that the evaluation of papilla and ONSD using transorbital sonography can be considered as a diagnostic tool for increased ICP in patients with IIH, while color doppler indices of ophthalmic vessels were not significant. Converesly, Jeub et al. (23) professed the usefulness of both transorbital B-Mode sonography and color doppler imaging of the central retinal artery in the IIH diagnosis, especially peak systolic velocity, which was increased in patients with IIH compared to controls. Furthermore, the authors found this parameter to be decreased to normal values after therapeutic LP, probably because increased pressure in the optic nerve caused a vessel's restriction (23).

### ONSD as Indirect Parameter to Assess Treatment Efficacy in Patients With IIH

Optic nerve sheath diameter has also been used to monitor the treatment efficacy in patients with IIH.

Lochner et al. (24) performed an ONSD sonography assessment in a female patient with IIH before and after LP and 12 months of treatment with acetazolamide (500 mg × 2) and diet to lose weight. Following LP, there was a bilateral decrease of ONSD, with unchanged optic disc elevation values, which completely normalized after 12 months of therapy.

In another study, Lochner et al. (25) performed ONSD ultrasound appraisal in overweight patients with IIH before and 6 months after starting different therapies: all patients were invited to follow a diet to lose weight, 17 patients were treated with acetazolamide, 3 were treated with topiramate, only 1 patient received topiramate and acetazolamide, and 4 received no medication. Six months later, ultrasound parameters and also symptoms, especially headache, significantly decreased.

Knodel et al. (26) published a case-control study enrolling 25 patients with IIH and 19 healthy controls and performing ONSD ultrasound evaluation at baseline and after 6 months of therapy (5 patients treated with only acetazolamide, 3 patients treated just with diet, 13 patients treated with acetazolamide and diet, 2 patients treated with topiramate and diet, 1 patient treated with acetazolamide and furosemide, 1 patient underwent shunt surgery), confirming how transorbital sonography is a valuable and non-invasive method to detect and monitor elevated ICP in IIH, as previously demonstrated by Lochner et al. (24, 25).

Sinclair et al. (27) noticed a reduction of ICP, ONSD, papilledema, and symptoms 3 months after a low energy diet to lose weight in 20 obese women with IIH. According to these outcomes, they suggested that weight loss could be an effective treatment in IIH and, consequently, a balanced low-calorie diet must be recommended in obese patients with IIH.

### ONSD and MRI Pituitary-to-Sella Height Ratio (Pit/Sella) in Patients With IIH

Patterson et al. (28) compared the performance of B-scan ultrasonography and MRI in detecting increased ICP in patients with IIH. Specifically, they measured ONSD using B-scan and axial T2-weighted MRI examination, calculating pituitary-to-sella height ratio (pit/sella) from sagittal T1-weighted MRI images to quantify the “empty sella” sign, frequently present in patients with IIH. They concluded that the specificity for IIH diagnosis can be improved by combining ONSD measurements and pit/sella ratio.

### A-Scan Ultrasonography in Patients With IIH

A-scan ultrasonography was utilized to measure optic nerve diameters (ONDs) by Salgarello et al. (29).

They performed standardized A-scan echography of the mid orbital ONDs and automated threshold perimetry (Humphrey 30-2) in 20 patients with IIH, comparing the results with 20 age-matched healthy controls. They found a significant increase of mean OND value in patients with IIH compared with the control group (mean right eyes 4.63 mm; mean left eyes 4.73 mm; mean controls 3.7 mm; *p* < 0.01); with ophthalmoscopic examination, bilateral papilledema was detected in 10 patients, unilateral papilledema in 1 patient, and normal optic disc in 9 patients. Regarding perimetric indices, there was a significant reduction in mean perimetric sensitivities, with a significant increase of mean deviation (MD) and pattern SD (PSD) in both eyes of patients with IIH, compared with normal age-corrected values. The correlation between perimetric indices and the corresponding OND values in both eyes of patients with IIH showed changes in MD and PSD in patients with papilledema and a correlation with OND values: the greater the transverse ONDs was, the fewer the Humphrey MD values and the greater PSD values were. These outcomes suggested that IIH functional alterations could be correlated to the degree of optic nerve sheaths enlargement, as a consequence of the rise of CSF pressure.

The main findings of the articles related to LP are shown in the Table 1, while the reference range values for IIH established in the other discussed articles are summarized in the Table 2.

**Table 1:** Summary of the optic nerve sheath diameter (ONSD) ultrasound reference range values established in studies on people with idiopathic intracranial hypertension (IIH) before and after lumbar puncture.

**Study**	**No. IIH patients**	**ONSD reference range values (mm)**	
		**ONSD before LP**	**ONSD after LP**
Singleton et al. (15)	1	RE: 7.20	RE: 5.8
		LE: 6.8	LE: 6.2
Hassen et al. (16)	1	7.8	5.7
Bäuerle et al. (17)	10	RE: 6.4 ± 0.6	RE: 5.8 ± 0.7
		LE: 6.4 ± 0.6	LE: 5.9 ± 0.7
del Saz-Saucedo et al. (18)	19	6.76 ± 0.61	Not reported
Kishk et al. (19)	99	6.57 ± 1.02	Not reported
Hassen et al. (21)	1	RE: not reported	RE: not reported
		LE: 7.0	LE: 4.1
Lochner et al. (24)	1	RE: 6.8	RE: 5.4
		LE: 6.6	LE: 5.4
			(measurements performed after LP and after 12 months of treatment with acetazolamide and diet)

*IIH, idiopathic intracranial hypertension; ONSD, optic nerve sheath diameter; SD, standard deviation; LP, lumbar puncture; RE, right eye; LE, left eye*.

**Table 2:** Summary of the ONSD ultrasound reference range values established in other studies on people with IIH.

**Study**	**No. IIH patients**	**ONSD reference range values (mm)**		
		**Mean ±SD**	**Min**	**Max**
Huo et al. (20)	5	Not reported	Not reported	Not reported
Ebraheim et al. (22)	24	At baseline:	Not reported	Not reported
		6.7 ± 0.5		
		After 1 week:	Not reported	Not reported
		6.6 ± 0.5		
		After 4 weeks:	Not reported	Not reported
		6.4 ± 0.6		
Jeub et al. (23)	19	RE 6.5 ± 0.2	RE 4.9	RE 8.2
		LE 6.6 ± 0.1	LE 5.7	LE 8
Lochner et al. (25)	22	At baseline:	Not reported	Not reported
		Median 6.51		
		After 6 months of diet and therapy:		
		Median 6.08		
Knodel et al. (26)	25	At baseline:	4.8	7.8
		6.2 ± 0.73		
		After 6 months of diet and therapy:	4.4	7.5
		6.0 ± 0.7		
Sinclair et al. (27)	25	At baseline:	Not reported	Not reported
		Not reported		
		After 3, 6 and 9 months of diet:		
		Not reported		
Patterson et al. (28)	44	Papilledema group (22):	Not reported	Not reported
		US-measured ONSD 5.2 ± 0.6		
		MRI-measured ONSD 7.2 ± 1.6		
		Pseudopapilledema group (22):		
		US-measured ONSD 4.4 ± 0.7		
		MRI-measured ONSD 5.2 ± 1.4		
Salgarello et al. (29)	20	RE 4.63 ± 0.84	Not reported	Not reported
		LE 4.73 ± 0.77		

*IIH, idiopathic intracranial hypertension; ONSD, optic nerve sheath diameter; SD, standard deviation; RE, right eye; LE, left eye*.

## Discussion

In recent years, ocular ultrasonography has widely spread because of its non-invasiveness, speed of execution, and quick obtainable results useful in the diagnosis of different diseases, such as IIH (30). Indeed, in all the articles evaluated in this review, transorbital ultrasound proved effective in the diagnosis of increased ICP and even in follow-up evaluation of patients with IIH.

Moreover, in all the discussed articles, a B-mode probe with a 7–13 MHz frequency was used, excepting Bäuerle et al. (17), using a 3–9 MHz linear array transducer, and Knodel et al. (26), using a 3–11 MHz one. Ultrasound assessments have been performed on subjects placed in the supine position with the head elevated to 20°–30°. ONSD measurements were taken at 3 mm posterior to the retina, at the edge of the optic nerve sheath, as the distance between the external margins of the hypoechogenic subarachnoidal space, which is surrounded by the dura mater (hyperechogenic) and periorbital fat. Two or three measurements have been taken from each eye. There is no agreement on the cut-off value for diagnosing raised ICP, with very different ONSD cut-off ranges.

B-scan ultrasound is probably the most diffused technique chosen in clinical practice, and even if it is considered a highly sensitive tool in the diagnosis and evaluation of patients with IIH, it is well known that the use of B-scan sonography has some limitations (31). In fact, there is the risk of having error in optic nerve diameter and ONSD assessment using the B-scan technique, due to the “blooming effect” consisting of a difference in the size of measured structures depending on the sensitivity settings (32, 33).

Conversely, the standardized A-scan technique is not affected by the blooming effect, giving the possibility to obtain more precise ONSD measurements, thanks to the noticeable high reflective arachnoid spikes (34, 35).

The only article discussed in the present review in which authors used A-scan ultrasonography has been published by Salgarello et al. (29). They measured OND on 20 patients with IIH with a documented increase in CSF pressure, using a mini A-standardized echographic probe (Biophysics Medical) and choosing a procedure already described in the literature by Ossoinig et al. (6, 7). Ultrasound measurements were taken with the open eye in the primary position and the optic nerve diameter corresponded to the distance between two spikes, each one coinciding to the arachnoid surrounding the optic nerve.

In addition to its blooming effect absence, the standardized A-scan technique has the advantage of allowing the use of the “30 degrees test,” which is a dynamic A-scan test that measures the width of the optic nerve in primary gaze and again when the patient shifts the gaze 30° from primary position (36). In cases of increased ICP, the nerve and sheath are stretched as the globe turns temporally, resulting in a shorter measurement value compared to the primary gaze. On the other hand, in the case of solid lesions, the ONSD will appear enlarged but the measurement does not change between the primary gaze and 30° (37, 38). Nonetheless, this is a more complicated test, taking considerable expertise, and not all ultrasound devices have the technological capability (39).

In conclusion, in the case of IIH, the results of our review show that ocular ultrasound has been widely used to detect and to follow up with these patients. B-scan offers the benefit of being quick and transportable and could be used by different specialists after a specific training (40). Unfortunately, B-scan has several limitations such as the blooming effect and artifacts that can make the measurements unreliable and poorly reproducible (41). For this reason, the use of standardized A-scan, which provides the best possible accuracy, is recommended for the diagnosis and during the follow-up, when the maximal care in measurements is needed (42).

## Author Contributions

MD, LV, ID, and LC analyzed the literature and wrote the original draft. PC and NR conceived the article and reviewed the manuscript. All authors read and approved the final manuscript.

## Funding

This research was funded with the FARB Grant from the University of Salerno.

## Conflict of Interest

The authors declare that the research was conducted in the absence of any commercial or financial relationships that could be construed as a potential conflict of interest.

## Publisher's Note

All claims expressed in this article are solely those of the authors and do not necessarily represent those of their affiliated organizations, or those of the publisher, the editors and the reviewers. Any product that may be evaluated in this article, or claim that may be made by its manufacturer, is not guaranteed or endorsed by the publisher.
